# Educating correct ECG interpretation is a way to promote the satisfaction and competency the diagnostic service providers 

**DOI:** 10.22088/cjim.15.1.16

**Published:** 2024

**Authors:** Mehrdad Saravi, Amirmohammad Rezaei Majd, Mohammad Ali Qassemi Darzi, Yasaman Habibzadeh, Zeinab Hoseini Motlagh, Maryam Ghaemi-Amiri

**Affiliations:** 1 Department of Cardiology, School of Medicine, Mobility Impairment Research Center, Health Research Institute, Rouhani Hospital, Babol University of Medical Sciences, Babol, Iran; 2 Razi Hospital. Tehran University of Medical Sciences, Tehran, Iran; 3 Medical Education Development Student Committee, Babol University of Medical Sciences, Babol, Iran; 4 Education Development Center, Babol University of Medical Sciences, Babol, Iran; 5 Department of Medical Education Development, Education Development Center, Babol University of Medical Sciences, Babol, Iran

**Keywords:** Electrocardiography, Teaching methods, Diagnostic services

## Abstract

**Background:**

Given electrocardiogram (ECG) interpretation as one of the diagnostical challenges for medical students and health professionals, this research was carried out to present an experience of web-based teaching method and novel approaches used for training of ECG interpretation.

**Methods:**

This online program was conducted in three days. The main content of the class was taught during one hour, and after that, the teacher spent enough time for responding the asked questions. The components of a normal ECG and different changes that can occur in these waves were taught through clinical case-based scenarios using the web platform and Adobe Connect software. The participants' satisfaction was assessed with a 12-item questionnaire, and the short-term retention of ECG interpretation skill was examined by comparing the posttest scores with pretest.

**Results:**

A total of 224 individuals completed the course. Total satisfaction score was 53.05±6.98 (out of the maximum score of 60). Based on the results of the paired t test, the interpretation skill scores of the participants increased significantly from 2.5 ± 1.57 to 6.96 ± 1.89. (p<0.001, CI = -4.8 to- 4.11).

**Conclusion:**

This web-based nationwide training program provided a supplementary resource for ECG learning among medical students and health-care providers.

Electrocardiogram interpretation is one of the core skills in the educational curriculum of many courses of medical sciences, and competence of ECG interpretation is among the major educational challenges for medical students and health care providers working in different departments (1, 2). Much greater, the skills of physicians, assistants, and health staff in ECG analysis and accurate identification of its changes, the faster interventions can be conducted for patients and the more favorable treatment outcomes in patients' care can be expected (2). One of the new teaching modalities in medical sciences is case scenario-based teaching (3). In this method, scenarios from different clinical cases are presented, and at the same time, different ECGs related to each scenario are displayed (4-6). This approach can be helpful for better perception and learning in trainees. 

The occurrence of coronavirus disease (COVID-19) outbreak in Iran impacted different aspects of medical sciences education in this country (7, 8); the most important effect of this disease was postponement of practical and apprenticeship classes (7). Given more feasibility and availability of web-based teaching of clinical case scenarios for assistance of ECG learning in medical students and health-care providers, this study was carried-out to present a nationwide experience of this teaching method and novel approaches used for training of ECG interpretation. 

Since ECG analysis has many complexities, different teaching techniques have been introduced in previous studies; some of them used traditional and face-to-face teaching methods, some recommended mobile or computer-assisted or web-based training, and others proposed blended teaching to improve ECG interpretation skills (1, 2, 9-11). Recent systematic reviews demonstrated more cost-effectiveness of digital platforms and web-based teaching for promotion of different medical skills such as ECG interpretation than that of traditional methods (10, 12).

## Methods

**Study design and setting: **This program was implemented on the platform of Adobe Connect with a daily capacity of 500 persons.This online educational program started since the first half of the academic year 2021-2022, by the Associate Professor of Cardiology (fellowship of electrophysiology studies) in collaboration with the Education Development Center (EDC) of Babol University of Medical Sciences, Iran. Medical sciences students and health-care providers such as specialized medical assistants; students or graduates of doctorates or masters in medical fields from all parts of the country were invited to participate in this educational program. Various means (including sending invitation letters to different medical schools and universities, announcement through virtual networks and social groups of medical students, etc.) were used to inform these target groups about time of holding the program and how to participate. The presenting poster of the program was distributed physically and electronically at different provinces of the country. Social networks such as WhatsApp, Telegram and Instagram were used for this purpose.

**The tutorial:** The training classes were held in three separate days; every day the program started at 6 pm. The main content of the class was taught during one hour, and after that, the teacher spent enough time (30-45 minutes) for responding the asked questions. The components of a normal ECG (P, QRS, and T waves) and different changes that can occur in these waves were taught online in clinical case-based scenarios using the web platform and Adobe Connect software. The participants could ask any question about each of the presented cases. Some new ideas were used in this program; for example, fluent expression of the teacher for presentation of complex concepts of ECG analysis; reading poems from the Hafez Shirazi Poetry book by the teacher, according to the coincidence of the second day of the program with the Iranian Yalda cultural ceremony; using animations and visual appeal in the content of the teaching slides; presentation of histological and pathological features about different clinical cases mentioned in the slides, bidirectional dialogue between teacher and trainees during the program; and explaining common diagnostic mistakes in ECG interpretation. Participation in the program was free of charge. Also, the program was held in the evening (not morning) hours to facilitate the attendance of more students and health-care staff. 

**Variables and assessment tools: ** Participation in all three days of the program was considered as completing the course. Gender and educational level of the participants was recorded in the research datasheet. A 12-point researcher-made questionnaire was used to assess the participants' point of view and their satisfaction about this program. To confirm the validity, valid methods of calculating the validity, i.e. (Content Validity Ratio) CVR were used to quantify the experts' opinions and CVI (Content Validity Index) was used to confirm the feasibility of the questionnaire. According to the statistics, CVR = 0.85 and CVI = 0.80 for the said questionnaire was confirmed in terms of validity. The face validity of the questionnaire was examined by the experts in the field of medical education, and its reliability was calculated using Cronbach's alpha coefficient of 0.92.Based on the Likert scale, each question had five options with a score of 1 to 5 for the very low, low, medium, high and very high satisfaction. Total score was in the range from 12 to 60 (the average score of 36). Also, pre-post exams were conducted for evaluation of the ECG interpretation skills of the participants, and the effect of this training program on their skills. The participants were invited to participate in these exams, both before the 1^st^ and after the 3^rd^ class they attended. In this exam, 10 common clinical cases with their ECG rhythm strips were presented. The number of correct and incorrect answers of the participants was counted to determine if there are any changes that could be attributed to this training program. 

**Statistical methods:** The collected information was analyzed using SPSS-17 software package. Independent t-test and one-way ANOVA were used for data analysis. A p-value less than 0.05 was considered as the significance level. The results of the test were checked by the research group, who were experts in Medical Education. This study verified by ethical code: 1400-259 in Babol University of Medical Sciences.

## Results

A total of 427 persons from different parts of the country participated in one of the three sessions of the educational sessions. Out of them, 224 (52.6%) individuals completed the course, all of them (224, 100%) delivered the satisfaction assessment questionnaire, and 224(, 100%) filled out the pre-post exams. Based on the results of the paired t test, the interpretation skill scores of the participants increased significantly from 2.5 ± 1.57 to 6.96 ± 1.89. (p<0.001, CI = -4.8 to -4.11).

The participants who completed the program 123 (61.5%) were females, and 101 (38.5%) were males. Most of them were general medicine students or general practitioners (MD) (107 persons, 47.7%); 103 (46.9%) were students or graduates of bachelor science (Bc), 8 (3.1%) were residents or PhD students, and 6 (2.3%) were Master of Science (MSc) students or graduates. The participants' satisfaction points have been summarized in the table 1. This table shows that in 11 out of the 12 items, the choice of "very high" satisfaction level was the most frequent option; only in one question (the trainee' s opinion about his/her learning in this program) the choice of "high" was selected with higher frequency than the "very high" option. Furthermore, in all items, the average score was higher than 4, and this score is attributed to the "high" satisfaction level. Total satisfaction score was calculated as 53.05±6.98. 

The association of total satisfaction score with gender and level of education of the participants has been presented in table 2. No significant association was found between total satisfaction score and gender (P=0.632) or level of education (P=0.927).Mean number of correct responses of the trainees in interpreting the ECG of the patients before the program was 5.24± 2.04 and improved to 5.42±2.06 after the course. The analysis of pre-post exams has been presented in table 3). 

**Table 1:** Descriptive measures of the participants' satisfaction points

**Questions**	**Distribution of the answers** **Number (Percent)**					**Mean±SD**
	**very low satisfaction**	**low satisfaction**	**medium satisfaction**	**high satisfaction**	**very high satisfaction**	
1. Was the content of this program compatible with your educational needs?	0	2 (0.9)	24 (11.1)	67 (29.8)	131 (58.2)	4.45±0.73
2. How was your learning in this program?	2 (0.9)	6 (2.7)	56(25.3)	86 (38.2)	74 (32.9)	4.00±0.87
3. Was the time of holding the program suitable for you?	1 (0.4)	7 (3.1)	32 (14.7)	70 (31.1)	114 (50.7)	4.28±0.86
4. Was the number of classes suitable?	4 (1.8)	14 (6.23)	43 (19.6)	56 (24.9)	107 (47.6)	4.10±1.03
5. How much was participation in this program valuable for you?	0	1 (0.4)	25 (11.4)	46 (20.4)	152 (67.6)	4.55±0.71
6. How much are you satisfied about the online implementation of this program?	0	3 (1.3)	24 (11.1)	64 (28.4)	133 (59.1)	4.45±0.74
7. Your opinion about the content of the presentation slides?	0	0	9 (4.4)	49 (21.8)	166 (73.8)	4.69±0.55
8. Your opinion about the teacher's respondence to the asked questions?	1 (0.4)	3 (1.3)	14 (6.7)	63 (28.0)	143(63.6)	4.52±0.71
9. Your opinion about the teacher's skill in presenting the contents?	0	2 (0.9)	11 (5.3)	56 (24.9)	155 (68.9)	4.61±0.63
10. Your opinion about the quality of the taught points?	0	2 (0.9)	12 (5.8)	57 (25.3)	153 (68.0)	4.60±0.64
11. Your opinion about the efficiency of this program on your clinical skills?	2 (0.9)	3 (1.3)	33 (15.1)	70 (31.1)	116 (51.6)	4.31±0.84
12. Your general satisfaction about this program?	2 (0.9)	0	20 (9.3)	61 (27.1)	141(62.7)	4.50±0.73

**Table 2:** Association of total satisfaction score with the participants' gender and level of education

**Demographic characteristics**		**Total satisfaction score** **Mean±SD**	**P-value**
**Gender**	Male (n=101)	52.79±6.80	0.632
	Female (n=123)	53.22±7.11	
**Level of education**	Bachelor of science (n=103)	52.83±7.30	0.927
	Master of science (n=6)	53.83±5.63	
	General medicine student or general practitioner (n=107)	53.16±6.81	
	PhD or residency (n=8)	54.25±6.34	

**Table 3:** Comparing the average skill of interpreting the electrocardiogram of Participants in Pre-test & Post-test

**CI**	P-value*	**Post-test**		**Pre-test**	
		**Std.Error Mean**	**Mean ±SD**	**Std. Error Mean**	**Mean ±SD**
-4.80 to -4.11	≤ 0.001	1.89	6.96	1.57	2.50

Paired t test *

## Discussion

This nationwide online ECG interpretation educational program which carried out with some new ideas of teaching resulted to positive satisfaction points of medical students and health-care providers; also, the correct answers of the trainees about the ECG analysis improved in post-survey compared to the pretest. 

The advantage of online teaching programs, especially during the COVID-19 pandemics has been studied in several studies; trainees can stay at home, can learn at their own pace and comfortable surroundings, and they have continuous access to training materials, although, lack of exposure to the real patient is one of the disadvantages of this type of training (13, 14). Viljoen et al. in a recent systematic review and meta-analysis examined whether computer-assisted ECG interpretation teaching is more effective than face-to-face teaching method in acquiring ECG competence among medical students and residents, and concluded that computer-assisted method in a blended teaching context is more effective than face-to-face teaching alone, especially if practical activities in addition to effective feedback to the trainees are provided (1). 

In Waechter's study, 314 medical students practiced different ECG rhythm strips through freely accessible online modules, and received continuous formative feedbacks to determine if they met the competency standards defined by their school; 97% of the participants indicated that the learning modules were effective and efficient; 92% reported it as an enjoyable teaching technique; 99% believed practice is required to learn ECG analysis, and 95% represented that immediate feedback was helpful (5). In this study, the participants' satisfaction about different aspects of the program, including its compatibility with their educational needs, time and how to implement, the content of the classes, the teacher's skill in teaching and responding to the trainees' questions, and their viewpoints about the efficiency of this program on their clinical competence was in high level. These positive points might be attributed to the study design, and new approaches applied for implementation of this program. These approaches are discussed here:

1. The teacher explained complex concepts of ECG strips with simple and fluent descriptions, besides the presentation of related clinical scenario, and histopathological features. Similar to our finding, Seif et al. recommended an integrated basic and clinical approach for promotion of knowledge and skill in ECG interpretation for medical students. They proposed this approach for better engagement and critical thinking of the trainees along with diagnostic and clinical reasoning of the students (15). Krasne et al. conducted an online ECG perceptual and adaptive learning module for teaching ECG interpretation to medical students and emergency medicine residents, and recommended this approach as an efficient supplemental tool for developing mastery in analysis common ECG abnormalities (16). In our study, an associate professor with more than 20 years of experience in the field of cardiology taught the program content in all three days and responded to the questions of the trainees, and this can have positive impact on the research outcomes.
2. Reading poems from a famous Iranian poet by the teacher in this program encourages new perspectives for consideration of culture in medical education (17-19). Also, reading poems facilitated emotional talks and helped make the learning environment more fine and non-boring.
3. Using animations and visual appeal in the content of the presenting slides might make the classes more exciting and attractable (20, 21). Recent evidence has demonstrated the effect of medias used in slide presentations on the communication between the instructor and trainees, and can be helpful for better transmission of educational messages to the trainees (22).
4. Providing an effective interaction between the participants and the teacher, responding to the trainees' questions, and explaining common diagnostic mistakes in ECG interpretation provided an appropriate condition for different trainees, including students and health-care staff to ask different questions in the field of ECG analysis, and receive good feedbacks from the instructor (23, 24).
5. Participation of medical students and graduates of medical sciences in this program provided a suitable environment for presenting frequently clinical questions the trainees were faced on in their work, including in schools, clinics, hospitals, and health service centers; and responding to these questions resulted to better educational outputs.

In our research, satisfaction score did not differ between men and women, also, no significant association was found between satisfaction score and the participants' level of education. Similar to our finding, Rolskov Bojsen et al. found no significant difference between male and female, and junior or senior medical students who participated in a web-based ECG training classes (6). Eysenbach showed that to use or not to use a web-based ECG training resource is based on multifactorial aspects such as experiences during clinical rotations, their previous experiences, and perceived learning needs (25); and this can justify the similar findings between men and women, and level of education of the trainees. 

The most important strong points of this study are its implementation at a nationwide extent, and invitation both students and graduates of medical sciences. We measured short term retention of ECG interpretation skill. This can be mentioned as a limitation of this study, and we cannot extrapolate the findings to long-term retention of this skill. 

This web-based nationwide training program provided a supplementary resource for ECG learning among medical students and health-care providers. Continuation of such programs with considering new educational ideas at regular and scheduled intervals is recommended. 
